# High fidelity defines the temporal consistency of host-parasite interactions in a tropical coastal ecosystem

**DOI:** 10.1038/s41598-020-73563-6

**Published:** 2020-10-08

**Authors:** V. L. Lopes, F. V. Costa, R. A. Rodrigues, É. M. Braga, M. Pichorim, P. A. Moreira

**Affiliations:** 1 Programa de Pós-Graduação em Ecologia de Biomas Tropicais, Universidade Federal de Ouro Preto – UFOP, Ouro Preto, Minas Gerais Brazil; 2 Departamento de Biodiversidade, Evolução e Meio Ambiente, Universidade Federal de Ouro Preto – UFOP, Campus Morro do Cruzeiro, Ouro Preto, Minas Gerais 35400-000 Brazil; 3 Departamento de Parasitologia, Universidade Federal de Minas Gerais – UFMG, Belo Horizonte, Minas Gerais Brazil; 4 Laboratório de Ornitologia, Departamento de Botânica e Zoologia, Universidade Federal do Rio Grande do Norte – UFRN, Natal, Rio Grande do Norte Brazil

**Keywords:** Ecological networks, Evolution, Parasite biology

## Abstract

Host-parasite interactions represent a selective force that may reduce hosts’ lifespan, their reproductive success and survival. Environmental conditions can affect host-parasite communities, leading to distinct patterns of interactions with divergent ecological and evolutionary consequences for their persistence. Here, we tested whether climatic oscillation shapes the temporal dynamics of bird-haemosporidian associations, assessing the main mechanisms involved in the temporal dissimilarity of their interactions’ networks. For two years, we monthly sampled birds in a tropical coastal ecosystem to avian malaria molecular diagnosis. The studied networks exhibited high specialization, medium modularity, with low niche overlap among parasites lineages. Moreover, alpha and β-diversity of hosts, parasites and their interactions, as well as the structure of their networks were temporally consistent, i.e., stable under fluctuations in temperature or precipitation over seasons. The structure and temporal consistency of the studied antagonistic networks suggest a high fidelity between partners, which is likely relevant for their evolutionary persistence.

## Introduction

Parasites encompass 40% of described species worldwide^1^, being able to exert an important selective pressure on their hosts. They can shape hosts community structure^2,3^, affecting their survival, reproductive success and behavior^4–6^. For instance, it is known that haemosporidian parasitism strongly affect avian-hosts community structure, including decline and extinction of hosts populations^7,8^. In this way, host-parasite interactions are excellent models for understanding the structure and temporal dynamics of antagonistic systems, such as emerging infectious diseases^9,10^.

Avian malaria is a vector-borne disease caused by globally distributed parasites of two genera, *Plasmodium* and *Haemoproteus*^11,12^, which infect a wide range of bird species^13^. Haemosporidian host specificity is variable, ranging from a unique host to many unrelated infected species^11,14,15^. Haemosporidian parasites are as diverse as their hosts, wherein regions with high bird richness also hold high parasite richness, emphasizing the importance of tropical ecosystems as major reservoirs of haemosporidian lineages^16^. As evidence suggests that in the tropics, the selective pressure of parasites is stronger than in temperate regions^17,18^, we might expect more stable populations^19^ and higher specialization towards tropical regions^20,21^. However, little disparity has been found between antagonistic avian-parasite systems^22^, as well as in host-parasitoid networks^23^ when comparing tropical and temperate regions.

Exploring ecological interactions by network approach help us to understand patterns of specialization and intimacy between interacting partners^24–27^. Recent studies have shown that the structure of antagonistic networks is influenced by ecological (e.g., climatic conditions) and evolutionary factors (e.g., phylogenetic relationships)^26–30^. Specifically for avian malaria infections, hosts characteristics (i.e., local abundance) and functional traits (i.e., body condition, sex, and feeding behavior) may contribute to parasites prevalence, affecting their communities’ organization and consequently the entire network structure^31–36^. Moreover, recent evidence suggests a lack of correlation between host specificity and haemosporidian prevalence, despite the strong network modularity, wherein modules represent phylogenetic proximity among host species^37^. As follows, it seems that modularity in interactions networks, i.e. a structure that emerges when cohesive subgroups of species interacts among themselves in higher frequency than with the remaining network^38^, is an important property that provides stability in antagonistic systems^29^.

Generally, host-parasite interactions comprise a high degree of intimacy and adaptation between partners^39,40^. For instance, ecological and phylogenetically related host species can promote network specialization in a way that their proximity (e.g., phylogenetic, ecological, or functional) is higher among species within the same network compartment or module^40–43^. This high affinity and coevolution of species ensure their continued association throughout time and space^44^. However, the insertion or deletion of new species and individuals can alter the temporal dynamic of these systems, with broader impacts on populations of either parasites or hosts^45^. Likewise, host specificity can vary within a species according to their geographic range, host community composition, and environmental conditions^10^. Hence, the temporal variation in species loss, species gain, and/or species turnover is determinant for understanding the transmission, infection and their dynamics^46^. In spite of advances, some aspects of host-parasites networks remain unexplored, such as their temporal dynamics over seasons^22,37^.

The scenario is even worse if we consider that avian malaria is one of the most prominent and widespread vector-borne diseases in wild animals^47^. Therefore, unveiling how these interactions responds to oscillating abiotic conditions, i.e., temperature and precipitation, is useful to predict how ongoing global changes may affect the ecology and evolution of antagonistic systems. After all, we must comprehend the dynamism of these interactions through time, as the causes and consequences of any such dynamic patterns. Nevertheless, to unveil how interaction networks change over time is important to understand the assembly and disassembly of biological interactions and their persistence under ecological and evolutionary unpredictability^29,48^.

Few studies of avian haemosporidian in Neotropics have considered the temporal variation of abiotic conditions on the prevalence and parasite lineages composition. For instance, no difference was found in parasites lineages composition over seasons at Caribbean Islands^49^. Other study has found that the effect of seasonality on parasites prevalence is detectable only for few bird species at Brazilian tropical dry forests^50^. As follows, the temporal dynamics of haemosporidian infections might be shaped by host species turnover (e.g., arrival of migratory birds), as well as by interactions switching between co-occurring species^51^. Regardless of current knowledge, still is unknown how fluctuations in abiotic conditions might predict the temporal dynamics of bird-haemosporidian networks.

This study aimed at exploring the temporal dynamics of avian hosts, haemosporidian parasites, and their interactions assembly under climatic fluctuations across seasons. Over two years, we studied infected birds from a tropical coastal ecosystem and tested whether: (1) the temporal dissimilarity of birds and parasites is driven by species turnover or species gain/loss across seasons; (2) whether species turnover or interaction switching modulate the temporal dissimilarity of bird-parasite interactions; (3) whether the structure of avian-parasite networks are determined by temporal oscillations of abiotic conditions (i.e., temperature and precipitation oscillation over seasons); and (4) whether the networks formed by distinct parasite genera, *Plasmodium* and *Haemoproteus*, are similar in terms of structure and infected host species.

## Results

During the two years, we captured 1,803 birds and recorded 319 recaptures. From the captures, 1060 (59%) were sampled in the rainy season and 743 (41%) in the dry season. The most abundant bird species in the rainy season were the two migratory species Chilean Elaenia (*Elaenia chilensis* 23%), and creamy-bellied Thrush (*Turdus amaurochalinus* 11%), besides the local (non-migrant) bananaquit (*Coereba flaveola* 8%). In the dry season, the most abundant were the two local species white-lined tanager (*Tachyphonus rufus* 16%), and plain-crested Elaenia (*Elaenia cristata* 14%), besides the migratory *C. flaveola* (7%). Despite the assessments of avian malaria prevalence are not the goal of this study, it is important to regard that of the 1,803 birds analyzed for the presence of *Plasmodium* and *Haemoproteus*, 443 were positive, representing a prevalence of 25%. Seventeen of the 443 infected host individuals (3.8%) exhibited multiple infections, based on double peaks in the chromatograms, and then were removed from our dataset. The most infected bird species were *T. rufus* (28%), *E. chilensis* (9%), and *C. flaveola* (7%) (see Supplementary Table S1). Moreover, we have found a total *γ-*diversity of 69 bird species (potential hosts) and an average *α*-diversity of 46.5 bird species per season, from which only 26 species were infected. Both *α* and *β-*diversities of hosts did not vary with temperature or precipitation (Table 1). Even though *α* and *β*-diversities of birds were consistent over time, the existing temporal dissimilarity was mostly due to species turnover (87%), than to species gain and/or loss across seasons (i.e., nestedness = 13%).

**Table 1:** Generalized linear models results showing the effect of temperature and precipitation on alpha (*α*) and beta (*β)* diversities of avian hosts, parasites lineages, their interactions, and network proprieties assessed during two years in a coastal ecosystem at Northeastern Brazil.

Response variable	Distribution	Predictors	Deviance	d.f. residual	P-value
*α*-Birds	Quasipoisson	Temperature	0.0056	6	0.936
	Quasipoisson	Precipitation	0.0058	6	0.935
*β*-Birds	Gaussian	Temperature	0.0048	6	0.834
	Gaussian	Precipitation	0.0121	6	0.740
*α*-Parasites	Quasipoisson	Temperature	0.2550	6	0.757
	Quasipoisson	Precipitation	0.6994	6	0.618
*β*-Parasites	Gaussian	Temperature	0.8443	6	0.852
	Gaussian	Precipitation	19.698	6	0.349
*α*-Interactions	Quasipoisson	Temperature	0.1592	6	0.829
	Quasipoisson	Precipitation	2.6999	6	0.391
*β*-Interactions	Gaussian	Temperature	2.9268	6	0.856
	Gaussian	Precipitation	119.78	6	0.215
Modularity	Gaussian	Temperature	0.0081	6	0.687
	Gaussian	Precipitation	0.0696	6	0.210
Specialization	Gaussian	Temperature	0.0522	6	0.495
	Gaussian	Precipitation	0.0609	6	0.459
Niche overlap	Gaussian	Temperature	0.0694	6	0.225
	Gaussian	Precipitation	0.0838	6	0.175

We obtained a total of 151 good qualities sequences of parasite lineages. From these, 44 (29%) comprised the genus *Plasmodium* spp., 96 (63.5%) the subgenus *Haemoproteus* (*Parahaemoproteus*) spp., and 11 (7.5%) the subgenus *H*. (*Haemoproteus*) spp. Additionally, we have observed a total *γ-*diversity of 28 haemosporidian lineages, with an average *α*-diversity of 13 lineages per season. Of the 28 lineages, 18 corresponded to *Plasmodium* and 10 to *Haemoproteus*, being eight of them described for the first time (see Supplementary Table S2). Likewise, both *α* and *β-*diversity of parasites did not vary with temperature or precipitation (Table 1). The existing temporal dissimilarity of parasites also was mostly due to species turnover (87%), rather than lineages gain/loss over seasons (i.e., 13% of nestedness).

**Figure 1:**
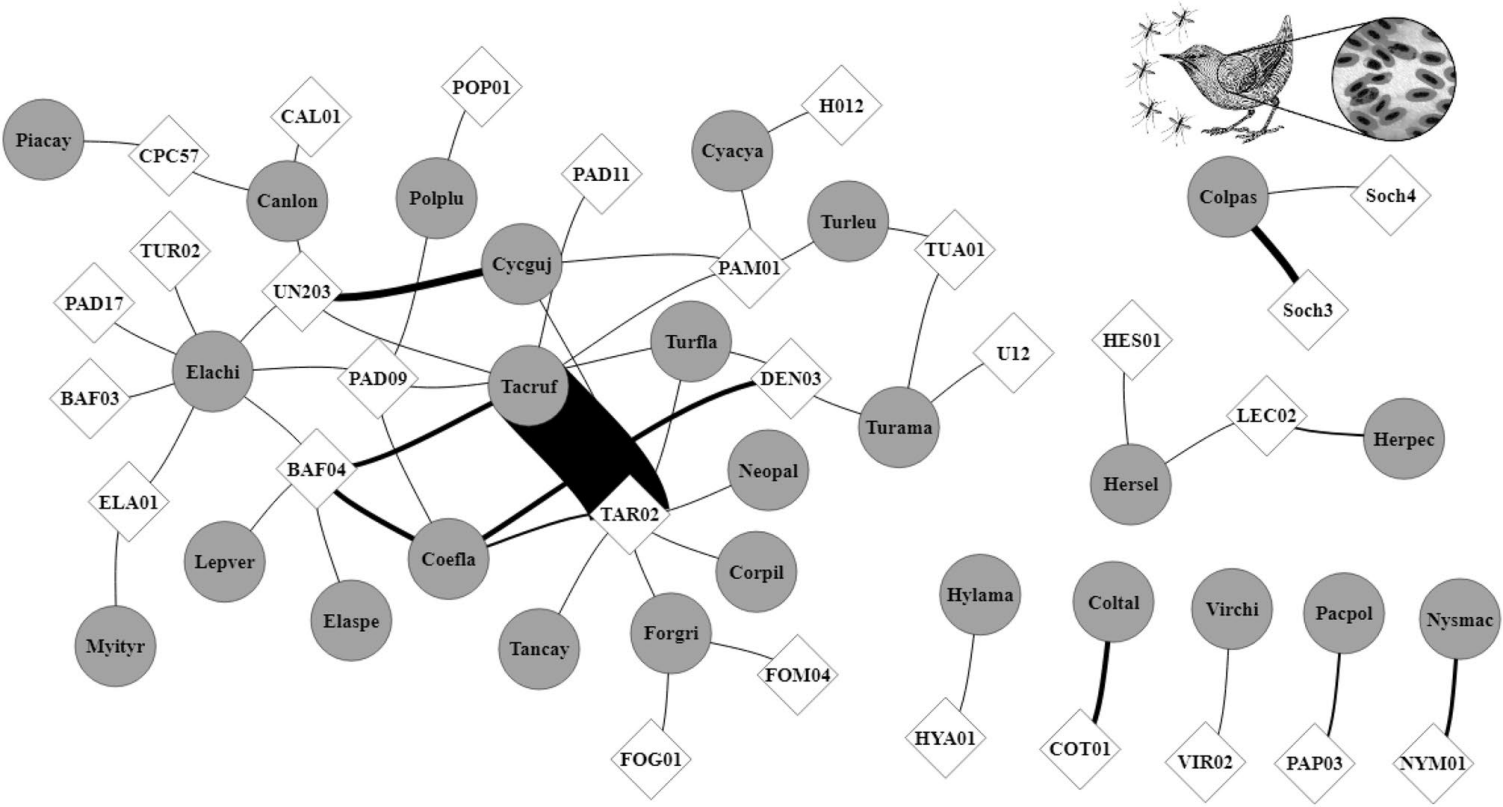
General network representing all avian-parasites interactions observed throughout the two sampling years in a tropical coastal ecosystem, Northeastern Brazil. Circles represent avian host species, diamonds represent parasite lineages, and lines thicknesses represent interaction frequencies. Parasite lineages and bird species codes can be found in Supplementary Tables S2 and S3, respectively.

Hence, out of the 69 potential hosts’ species, 26 were infected by 28 haemosporidian lineages (considering both parasite genera), totaling 141 host-parasite interaction events and 52 distinct interacting pairs (Fig. 1, see Supplementary Table S3). Among these, 64 interactions (45%) occurred in the rainy season and 87 (55%) in the dry season. Similarly, to hosts and parasites, *α* and *β-*diversity of interactions were not affected by temperature or precipitation (Table 1). Moreover, interactions’ pairwise comparisons (time to time) indicated that 43% of the temporal dissimilarity was due to the turnover of hosts and/or parasites species (${\beta }_{ST})$, while 57% was due to the rearrangement of interactions between species that temporally co-occur (${\beta }_{OS}$) (Supplementary Table S4).

In general, host-parasite networks presented medium modularity (Q = 0.53), high specialization (H_2_^′^ = 0.70), and low niche overlap among parasites lineages (Horn = 0.17) (see Supplementary Table S5). Nevertheless, modularity, specialization, and niche overlap did not vary with oscillations in temperature or precipitation, being consistent over time (Table 1).

**Figure 2:**
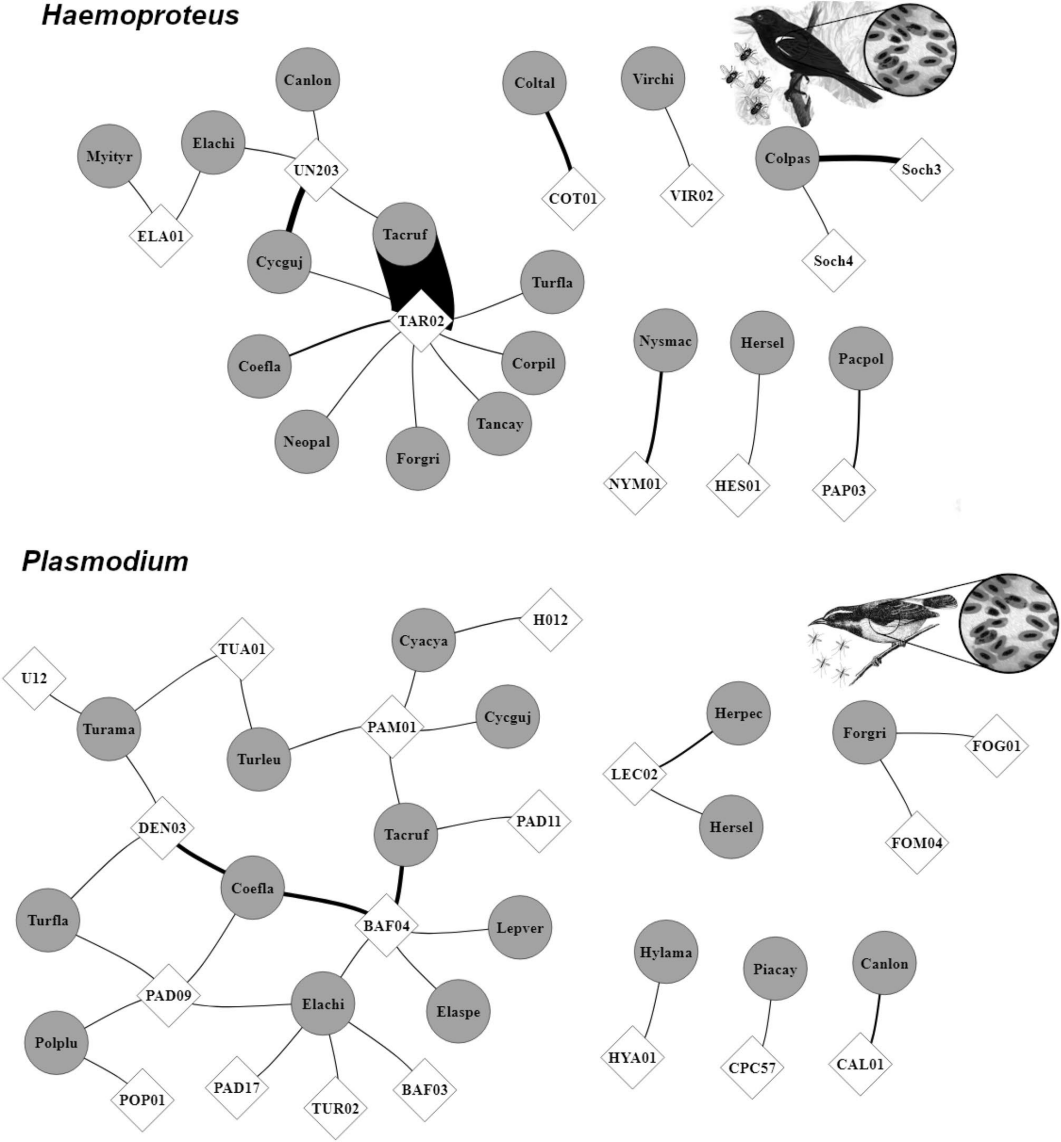
Interaction networks of *Haemoproteus* and *Plasmodium* parasites detected during the two sampling years in a tropical coastal ecosystem, Northeastern Brazil. Circles represent the avian host species, diamonds represent parasite lineages, and lines thicknesses indicate the frequency of interactions. Parasite lineages and bird species codes can be found in Supplementary Tables S2 and S3, respectively.

To end, we have found that both studied parasite genera presented similar network structures, with medium modularity (Q = 0.40 for *Plasmodium* and 0.42 for *Haemoproteus*), high specialization (H_2_′ = 0.63 for *Plasmodium* and 0.88 for *Haemoproteus*), and very low niche overlap among parasites (Horn = 0.02 and 0.06, respectively). Despite similarities in networks’ structure, the central hosts’ species in each genus were distinct (Fig. 2). Regarding *Plasmodium*’s hosts, we observed the following exclusive central species: *Coereba flaveola* (21% of interactions’ frequency), *Elaenia chilensis* (12%), and *Turdus amaurochalinus* (7%) (Fig. 2). Conversely, common ground dove (*Columbina passerina*) and rufous-browed peppershrike (*Cyclarhis gujanensis*) (both with 7% of interactions’ frequency) were exclusive and central in *Haemoproteus* network (Fig. 2). Only *Tachyphonus rufus* was central in both genera networks, but with distinct pattern of interaction. This host was most infected by *Haemoproteus* (63% of interactions’ frequency), than by *Plasmodium* lineages (14% of interactions’ frequency). Similarly, we observed a considerable difference in central lineages when comparing parasites genera. Five lineages were central in *Plasmodium* network: BAFLA04 (26% of interactions’ frequency), DENPET03 (13%), PADOM09 (9%), PAMIT01 (9%), and LECOR02 (7%). *Haemoproteus* though, presented two central lineages: SocH3 (6%) and TARUF02 (responsible for 70% of interactions, mainly with *T. rufus*) (Fig. 2).

## Discussion

Why do ecological interactions vary over time? This is an intriguing and open question that try to disentangle how and why species interact^52^. Here, we have found strong evidence that antagonistic networks between avian hosts and haemosporidian parasites are temporally consistent due to high partner fidelity. Our findings indicate that hosts, parasites, and their interactions do not vary under temperature and precipitation oscillations, being stable over seasons. Furthermore, we observed that bird and parasite species turnover and interaction switching between co-occurring species (i.e., interaction rewiring) contributes similarly for the temporal dissimilarity of studied interactions. Below, we discuss possible ecological and evolutionary processes involved in the temporal consistency of the studied avian malaria system.

The *α* and *β*-diversities of parasites and hosts were constant under temperature and precipitation oscillation throughout seasons, indicating that both partners communities are likely adapted to the local abiotic conditions^53^. These findings corroborate recent evidence that suggest that birds’ *β*-diversity is not dependent on environmental conditions, even facing pronounced climatic oscillations^54^. In addition, we observed that the temporal dissimilarity of avian hosts and parasites is largely due to species turnover (rather than nestedness), which can be explained by the great influence of migratory bird species, such as *Turdus amaurochalinus* and *Elaenia chilensis* that visit the study area during rainy season. Even though migrants likely promote a temporal replacement of species in the interacting communities, their overall dynamics remain stable under seasonality.

The studied host-parasite networks exhibited high specialization, medium modularity and low niche overlap among parasites. Together these networks structural patterns indicate that the studied system is strongly specialized, i.e., some groups of species interact more closely with others that likely hold phylogenetic proximity and/or compatible functional traits that allow interactions’ intimacy^55^. In fact, specialization in host-parasites networks has been detected in an avian malaria system, in both tropical and temperate areas^22^. Modularity in host-parasite networks suggests that certain groups of parasites tend to infect specific hosts groups, increasing system compartmentalization and specialization^56^. This network propriety thus, is directly related to the ecological and/or evolutionary proximity among species pairs^41^, a pattern already documented for avian malaria^37^ and other antagonistic systems, such as fish-endoparasite^40,57^, reptile-endoparasite^58^, mammal-ectoparasite^59^, and lepidopteran herbivores-plants networks^29^.

The observed low niche overlap among parasite lineages evidences the lack of host sharing. It is likely that this pattern results from constraints in foraging habit, nesting substrate, among other characteristics involved in hosts’ exploitation^41^. High niche partitioning also suggests indirect effects of competition among parasites, which consequently restricts resource sharing and promotes high host fidelity^60^. Likewise hosts and parasites, all networks’ proprieties were consistent under oscillations in abiotic conditions across seasons (Table 1). Altogether, these results demonstrate the high cohesion of the studied interactions, which is maintained even with the huge arrival of migrant hosts that represent potential sources of infection. Indeed, at least 32% of sampled birds correspond to sampled migrant species, *Turdus amaurochalinus* and *Elaenia chilensis.*

The temporal dissimilarity of interactions was similarly shaped by species turnover and interaction rewiring between shared species in seasons. This result indicates that changes in species composition and interactions switching throughout time, similarly affect the dynamics of the studied system^61^. Most ecological network studies have been pointing that interactions’ rearrangement between co-occurring species may be the most prevalent and expected process that shape community assembly across space and/or time. Nevertheless, distinct mechanisms, such as local abundance, trait matching, and phylogenetic proximity may determine species interactions^52^, particularly considering specialized systems. Since pairs’ rearrangement and species turnover are both underlying the temporal dissimilarity of the studied avian malaria infections, we might suppose that temporal changes in host and/or parasite species does not necessarily imply in interactions switching. Therefore, the temporal dynamics of studied interactions may be predicted both by species turnover and interactions' rewiring, evidence not yet described for antagonistic networks.

The distinct parasite genera exhibited a similar pattern of network structure, i.e., high specialization and very low niche overlap among congeneric lineages. Nonetheless, the most infected hosts were distinct between them. The distribution and intensity of parasites infections are commonly related to biological, ecological, and/or phylogenetic aspects^28^. Functional proximity amid hosts’ feeding guilds has been described as an ecological mechanism for maintaining parasite lifespan, as well as networks assembly^30^. In fact, we observed that all central birds in *Plasmodium* network holds similar functional traits, as feeding behavior and nesting type, important matches already reported for avian malaria^32,34,62^. Besides trait matching, the central birds infected by *Plasmodium* are phylogenetically proximal (i.e., within Passeriformes order) and may exhibit similar immune defenses^63–65^. As well, the central hosts of *Haemoproteus* hold high intimacy with their parasites, which can be evidenced by isolated infections (e.g., the compartment formed by *Columbina passerine*, SocH3, and SocH4 lineages; Fig. 2). Haemosporidians of *Haemoproteus* genera are divided into two subgenera and these both lineages (SocH3 and SocH4) belongs to the subgenus *H. (Haemoproteus)* that infected birds of Columbiformes order (e.g., *C. passerine*)^11^. The other two central hosts in *Haemoproteus* network (i.e., *Tachyphonus rufus* and *Cyclarhis gujanensis*) belong to Passeriformes order and interact with phylogenetically close *Haemoproteus* lineages (UN203 and TARUF02; see Supplementary Fig. S1). Taken as a whole, these findings reinforce the prevalence of cohesive and stable nucleus of interacting species, a pattern commonly described for mutualistic networks^66,67^, but barely explored in antagonistic systems^29,42^.

For the first time, we revealed a constant turnover of avian hosts and haemosporidian parasites over time, even under the influence of migrant hosts. The studied antagonistic networks are highly specialized with the emergence of cohesive groups of partners that are stable under climatic seasonality. Additionally, we have brought that species turnover and interaction rewiring similarly contribute to predict the temporal dissimilarity of avian-haemosporidian interactions. Altogether, these findings point out to the temporal stability of the studied system that can be a key mechanism to the parasitism’s evolutionarily persistence^39^. Nonetheless, we must pinpoint that our study encompasses a limited number of observations that should be seen carefully in further comparisons. The understanding of proprieties that emerge from complex antagonistic systems, such as avian malaria, is urgent as it can be used as models to predict how emergent diseases can potentially impact the health of ecosystems.

## Material and methods

### Study area

The study was carried out at the Barreira do Inferno Rocket Launch Center of the Brazilian Air Force (Centro de Lançamento Barreira do Inferno – CLBI), located in Parnamirim, Rio Grande do Norte State, Brazil (5°55′30″ S—35°9′47″ W). The CLBI has approximately 1,800 hectares and is inserted in a coastal ecosystem in the Atlantic Forest domain, locally called *Restinga*. The vegetation is characterized by marine influence, occurring on coastal sandy deposits, with xerophilic vegetation cover and predominance of herbaceous and shrub species, as well as semideciduous lowland forests. As CLBI has restricted access, there is no evidence of logging or burning inside its limits.

The climate of the region is tropical As (according to Köppen’s classification), with dry summers and rainy winters^68^, mean temperature of 25.6 °C and annual mean rainfall of 1261 mm. From April 2013 to March 2015, we obtained climatological data of monthly average temperature and monthly accumulated rainfall from a weather station managed by the National Institute of Meteorology (INMET in Portuguese acronym, see Supplementary Fig. S2), which is located at approximately 18 km from the study site. Following these climatic data, we defined the rainy season, as the period that goes from March to August and the dry season, as the period from September to February; seasons wherein that are significant changes in temperature and precipitation^69^ (Supplementary Fig. S1).

### Sample design

Inside the study area, we delimited a quadrant of 350 × 350 m (ca. 12 ha), in which 49 sampling points were placed 50 m from one another. In each sampling point we installed a mist net (Ecotone 18 × 3 m, mesh 19 mm and five shelves) to capture the birds. In each sampling day, we started to open the mist nets approximately 30 min before sunrise (between 04h30 and 05h10), with subtle variations that eventually occur along the year. Thus, the nets stayed open for 5 h and were inspected every 30 min, with no sampling during rainy days. We sampled the birds monthly, between April 2013 and March 2015, totaling 24 temporal samplings. In each month, collections were performed for two consecutive days, in which 25 nets were assembled on the first day and 24 nets on the second day. Captured individuals were identified and marked with metal bands provided by the Research Center for Wild Bird Conservation (CEMAVE in Portuguese acronym). Afterwards, we collected blood from sampled birds by puncturing the brachial vein with a sterile needle (13 × 4.5 mm). The blood was packed in filter paper and stored at − 4 °C until DNA extraction.

Our use of mist-nets and banding at the fieldwork was approved by the Brazilian biodiversity monitoring agency (Institute Chico Mendes for Biodiversity Conservation—ICMBio, Brazilian National Center for Bird Conservation—CEMAVE, permission 3239). We followed standard ethical protocols for wildlife animals. Time in captivity was kept to a minimum, and all individuals were released at the same place they were captured. This study was approved by the Ethics Committee in Animal Experimentation (CETEA), Universidade Federal de Minas Gerais, Brazil (Protocol #254/2011).

### Molecular analysis and detection of host-parasite interactions

The parasites were detected by molecular identification of the haemosporidian lineages present in the blood of infected birds. The genomic material was extracted using the phenol–chloroform method^70^. DNA was used for molecular diagnosis of haemosporidian through Polymerase Chain Reaction (PCR) by amplifying a region highly conserved from the mitochondrial SSU rRNA gene using primers 343F (5′GCTCACGCATCGCTTCT3′) and 496R (5′GACCGGTCATTTTCTTTG3′)^71^. It is important to emphasize that the parasite gene of both *Plasmodium* and *Haemoproteus* were amplified in the same reaction. To detect the infection, we used a positive control and a negative control in each diagnostic PCR. The positive controls consisted of DNA extracted from blood samples of chickens that were experimentally infected with *Plasmodium gallinaceum*, and the negative controls were ultrapure water. PCR products were viewed on a 6% acrylamide gel^72^.

Infected individuals in avian malaria screening were subsequently submitted to a nested-PCR which amplifies a 524 bp fragment of the mtDNA cytochrome *b* gene. For the first amplification we used the primers HaemNFI (5′CATATATTAAGAGAAITATGGAG3′) and HaemNR3 (5′ATAGAAAGATAAGAAATACCATTC3′); and for the second amplification the primers were HaemF (5′ATGGTGCTTTCGATATATGCATG3′) and HaemR2 (5′GCATTATCTGGATGTGATAATGGT3′)^73^. This nested-PCR was used to identify parasites lineages and did not detect avian malaria parasitemia.

The amplification products were sequenced in both directions using the BigDye Terminator Kit v3 (Applied Biosystems, Foster City, CA, USA) using an ABI3730 automated sequencer (Applied Biosystems, Foster City, CA, USA), in order to identify the diversity of lineages associated with sampled avifauna. PCR products were purified using a solution of 20% polyethylene-glycol 8000^70^. The quality of electropherograms generated was verified in the Phred v. 0.20425 program^74^. The sequences were visualized and edited in the Consed 12.0 program, and alignment and final assembly of sequences was performed in the Phrap v. 0.990319 program. We compared the obtained sequences and deposited the ones that were identified for the first time in GenBank and MalAvi^75^ databases. Sequences with differences in one or more nucleotides were considered distinct lineages of cytochrome *b*. Sequences that showed double peak, making lineage identification impossible, were removed from the analyses.

### Data analysis

With the molecular detection of parasites lineages associated with the studied avifauna, we constructed adjacent matrices of host-parasite interactions records obtained over 24 months of sampling. First, we built a complete matrix containing all interactions observed over the two years. Then, in order to get a suitable number of replicates,eight distinct matrices were constructed with the interactions recorded in the early and late rainy and dry season of each year. These eight networks corresponded to eight distinct periods of sampling: early rainy season (March to May), late rainy season (June to August), early dry season (September to November), and late dry season (December to February) of each studied year. Lastly, we prepared two distinct matrices, one for each parasite genus. In these matrices, rows correspond to the host bird species *i* and the columns to the parasite lineages *j*. We filled the matrices with the number of events registered between each host *i* and parasite *j* (i.e., interaction frequency, excluding recaptures with the same host-parasite interaction).

To test whether the temporal dissimilarity of birds and parasites are driven by species turnover or species gain/loss (i.e. nestedness^76^) over periods, we calculated the alpha (*α*) diversity of birds and parasites, which corresponds to the number of species and lineages found in each period. The beta (*β*) diversity of birds and parasites were calculated using the multiplicative partitioning of diversity^77^: $\beta =\frac{\gamma }{\alpha }$, where the gamma diversity (*γ*) corresponds to the total number of species/lineages found in the two sampling years. Moreover, we decomposed the β-diversity of parasites and birds into species turnover or species gain/loss across seasons, to calculate the contribution of each diversity component to the temporal dissimilarity. In addition, to test whether *α* and *β*-diversity of birds and parasites are determined by temporal oscillations of abiotic conditions, we built Generalized Linear Models (GLM), wherein *α* and *β*-diversity were response variables and mean temperature and precipitation over seasons (i.e., early and late rainy and dry seasons) were predictor variables (each predictor fitted separately in a distinct model). Measurements of species *β*-diversity were calculated with the package *betapart*^78^ in R^79^.

To test whether species turnover or interaction switching (or rewiring) modulate the temporal dissimilarity of avian-parasite interactions, we followed the approach^61^: ${\beta }_{WN}={\beta }_{ST}{ + \beta }_{OS}$, in which ${\beta }_{WN}$ indicates the total *β*-diversity of interactions, which is calculated through pairwise comparisons of temporal host-parasite networks (e.g., early rainy season 1—late rainy season 1, early rainy season 1—early rainy season 2, and so on), and represents the dissimilarity between distinct times . ${\beta }_{ST}$ represents the dissimilarity of interactions due to species turnover across seasons, and ${\beta }_{OS}$ is the dissimilarity due to interactions rewiring between partners that co-occur in each period (i.e., switching between shared species). This formula is based on the dissimilarity measure, ${\beta }_{w}$^77^, defined as: ${\beta }_{w}=\frac{a+b+c}{(2a+b+c)/2}-1$, in which *b* are interactions found at a sampling time (i.e., one period), *c* are interactions found at another time (i.e., a distinct comparative period), and *a* are interactions found at both times. Thus, the temporal *β*-diversity of interactions between shared species, ${\beta }_{OS}$, consists of variations in networks due to the interactions rewiring^25,61^. All values range from 0 to 1, and ${\beta }_{OS}$ is always equal to or less than ${\beta }_{WN}.$ For example, when ${\beta }_{OS}$ equals 1, it indicates that all dissimilarity between the temporal networks, e.g., early rainy season 1 *vs.* late rainy season 1, is due to interactions rewiring. Measurements of interactions’ *β*-diversity were calculated with *betalink* package^61^.

To test whether avian-parasite networks are affected by temporal oscillations in abiotic conditions, we used three metrics that are very robust to differences in sampling effort and network size^80^, and widely used in ecological network studies: complementary specialization at network level (H_2_′), which indicates how intimate species associations are. Values closer to 0 indicate high generalization or redundancy of interactions, and values closer to 1 indicate high specialization^24^; modularity (Q) using QuanBiMo algorithm (Q-values ranging from 0—low to 1—high modularity), which reveals the establishment of groups of species that are more connected among themselves, than with the remaining network species^81^; and finally, niche overlap among parasites (Morisita-Horn index, values from 0 to 1) that indicates the degree of host sharing by parasites^82^. These metrics corresponded to the response variables in the GLM, while mean temperature and precipitation fluctuation across seasons corresponded to the predictor variables used to assess the temporal consistency of networks’ structure under climate oscillation (each predictor fitted separately in a distinct model). All network’s metrics and their significance against Patefield null models (n = 999 randomizations) were obtained with the *bipartite* package^24^.

To end, to evaluate whether the networks of distinct parasite genera are similar and to identify the most important host species associated with each group, we calculated the central bird species and parasite lineages. For each parasite genera, we identified the species that performed a higher frequency of interactions than network average^66^. All statistical analyses were performed in R^79^ and host-parasite interaction networks were built in Pajek program^83^.

## Supplementary information

Supplementary Information.

## Data Availability

The datasets generated during and/or analysed during the current study are available in the Supplementary Information files. The sequence identifiers of all parasites’ lineages deposited in GenBank can be found in Supplementary Table S2, further data are available from the corresponding author on reasonable request.
