# Supplementary material for: High fidelity defines the temporal consistency of host-parasite interactions in a tropical coastal ecosystem
Source: Sci Rep. 2020 Oct 8;10:16839. doi: 10.1038/s41598-020-73563-6 (PMC7545182; doi:10.1038/s41598-020-73563-6)
Supplement: Supplementary file 1 — Supplementary Information. [file 41598_2020_73563_MOESM1_ESM.docx]

**High fidelity defines the temporal consistency of host-parasite interactions in a tropical coastal ecosystem**

VL LOPES^1,2^, FV COSTA^1^, RA RODRIGUES^3^, ÉM BRAGA^3^, M PICHORIM^4^, PA MOREIRA^1,2*^

*^1^ Programa de Pós-Graduação em Ecologia de Biomas Tropicais, Universidade Federal de Ouro Preto – UFOP, Ouro Preto, Minas Gerais, Brazil.*

*^2^ Departamento de Evolução, Biodiversidade e Meio Ambiente, Universidade Federal de Ouro Preto – UFOP, Ouro Preto, Minas Gerais, Brazil.*

*^3^ Departamento de Parasitologia, Universidade Federal de Minas Gerais – UFMG, Belo Horizonte, Minas Gerais, Brazil.*

*^4^ Laboratório de Ornitologia, Departamento de Botânica e Zoologia, Universidade Federal do Rio Grande do Norte – UFRN, Natal, Rio Grande do Norte, Brazil.*

**Supplementary Information**

Supplementary Table S1. Bird species sampled during the two sampling years in a coastal ecosystem at Barreira do Inferno Rocket Launch Center - CLBI, Northeastern Brazil. The numbers inside parentheses correspond to the total infected individuals by species.

| **Order** | **Family** | **Species** | **Year 1** | | **Year 2** | | **Total** |
| --- | --- | --- | --- | --- | --- | --- | --- |
|  |  |  | **Rainy** | **Dry** | **Rainy** | **Dry** |  |
| Passeriforme | Parulidae | *Basileuterus culicivorus* | 0(0) | 1(0) | 4(1) | 1(0) | 6(1) |
| Passeriforme | Tyrannidae | *Camptostoma obsoletum* | 1(0) | 2(0) | 0(0) | 1(0) | 4(0) |
| Passeriforme | Troglodytidae | *Cantorchilus longirostris* | 7(2) | 6(4) | 15(3) | 4(1) | 32(10) |
| Passeriforme | Pipridae | *Chiroxiphia pareola* | 2(0) | 1(0) | 0(0) | 0(0) | 3(0) |
| Passeriforme | Tyrannidae | *Cnemotriccus fuscatus* | 5(1) | 12(0) | 10(0) | 4(0) | 31(1) |
| Cuculiformes | Cuculidae | *Coccyzus melacoryphus* | 1(0) | 1(0) | 0(0) | 0(0) | 2(0) |
| Passeriforme | Thraupidae | *Coereba flaveola* | 52(9) | 30(12) | 33(8) | 21(3) | 136(32) |
| Columbiformes | Columbidae | *Columbina passerina* | 5(3) | 18(9) | 3(0) | 2(1) | 28(13) |
| Columbiformes | Columbidae | *Columbina picui* | 0(0) | 1(0) | 0(0) | 0(0) | 1(0) |
| Columbiformes | Columbidae | *Columbina talpacoti* | 1(1) | 7(5) | 6(1) | 0(0) | 14(7) |
| Passeriforme | Thraupidae | *Coryphospingus pileatus* | 0(0) | 3(1) | 2(0) | 1(0) | 6(1) |
| Passeriforme | Thraupidae | *Cyanerpes cyaneus* | 0(0) | 1(1) | 0(0) | 0(0) | 1(1) |
| Passeriforme | Corvidae | *Cyanocorax cyanopogon* | 2(2) | 0(0) | 0(0) | 0(0) | 2(2) |
| Passeriforme | Cardinalidae | *Cyanoloxia brissonii* | 0(0) | 0(0) | 1(0) | 1(0) | 2(0) |
| Passeriforme | Vireonidae | *Cyclarhis gujanensis* | 7(5) | 5(5) | 7(3) | 5(0) | 24(13) |
| Passeriforme | Thraupidae | *Dacnis cayana* | 4(0) | 4(0) | 10(1) | 7(0) | 25(1) |
| Passeriforme | Dendrocolaptidae | *Dendroplex picus* | 0(0) | 2(0) | 0(0) | 0(0) | 2(0) |
| Passeriforme | Tyrannidae | *Elaenia chilensis* | 95(26) | 2(0) | 151(15) | 0(0) | 248(41) |
| Passeriforme | Tyrannidae | *Elaenia chiriquensis* | 0(0) | 0(0) | 1(0) | 2(0) | 3(0) |
| Passeriforme | Tyrannidae | *Elaenia cristata* | 37(8) | 67(16) | 38(1) | 34(1) | 176(26) |
| Passeriforme | Tyrannidae | *Elaenia flavogaster* | 4(1) | 3(1) | 2(0) | 2(1) | 11(3) |
| Passeriforme | Tyrannidae | *Elaenia spectabilis* | 1(1) | 3(0) | 4(1) | 2(0) | 10(2) |
| Passeriforme | Tyrannidae | *Empidonomus* | 0(0) | 0(0) | 1(0) | 0(0) | 1(0) |
| Passeriforme | Tyrannidae | *Empidonomus varius* | 0(0) | 0(0) | 1(0) | 0(0) | 1(0) |
| Passeriforme | Fringilidae | *Euphonia chlorotica* | 0(0) | 1(1) | 3(0) | 3(0) | 7(1) |
| Passeriforme | Tyrannidae | *Euscarthmus meloryphus* | 0(0) | 1(0) | 0(0) | 0(0) | 1(0) |
| Passeriforme | Thamnophilidae | *Formicivora grisea* | 4(2) | 4(1) | 7(2) | 6(1) | 21(6) |
| Galbuliformes | Galbulidae | *Galbula ruficauda* | 0(0) | 1(0) | 0(0) | 0(0) | 1(0) |
| Passeriforme | Rhynchocyclidae | *Hemitriccus margaritaceiventer* | 12(2) | 18(10) | 10(0) | 6(0) | 46(12) |
| Passeriforme | Thamnophilidae | *Herpsilochmus pectoralis* | 3(2) | 9(4) | 14(5) | 8(2) | 34(13) |
| Passeriforme | Thamnophilidae | *Herpsilochmus sellowi* | 5(2) | 10(2) | 14(2) | 10(0) | 39(6) |
| Caprimulgiformes | Caprimulgidae | *Hydropsalis torquata* | 8(2) | 9(4) | 16(2) | 2(0) | 35(8) |
| Passeriforme | Vireonidae | *Hylophilus amaurocephalus* | 5(2) | 17(6) | 12(3) | 10(1) | 44(12) |
| Columbiformes | Columbidae | *Leptotila verreauxi* | 1(0) | 2(0) | 3(1) | 4(0) | 10(1) |
| Passeriforme | Mimidae | *Mimus gilvus* | 0(0) | 0(0) | 3(0) | 0(0) | 3(0) |
| Passeriforme | Tyrannidae | *Myiarchus ferox* | 2(0) | 0(0) | 0(0) | 0(0) | 2(0) |
| Passeriforme | Tyrannidae | *Myiarchus tyrannulus* | 0(0) | 3(1) | 1(0) | 0(0) | 4(1) |
| Passeriforme | Tyrannidae | *Myiodynastes maculatus* | 1(0) | 0(0) | 0(0) | 0(0) | 1(0) |
| Passeriforme | Tyrannidae | *Myiopagis viridicata* | 0(0) | 1(0) | 0(0) | 0(0) | 1(0) |
| Passeriforme | Parulidae | *Myiothlypis flaveola* | 0(0) | 1(0) | 1(0) | 0(0) | 2(0) |
| Passeriforme | Pipridae | *Neopelma pallescens* | 3(1) | 3(0) | 14(2) | 7(0) | 27(3) |
| Caprimulgiformes | Caprimulgidae | *Nyctidromus albicollis* | 0(0) | 2(1) | 1(0) | 0(0) | 3(1) |
| Galbuliformes | Bucconidae | *Nystalus maculatus* | 2(1) | 1(1) | 6(1) | 2(2) | 11(5) |
| Passeriforme | Tityridae | *Pachyramphus polychopterus* | 1(1) | 3(2) | 0(0) | 3(0) | 7(3) |
| Passeriforme | Tyrannidae | *Phaeomyias murina* | 1(0) | 2(0) | 0(0) | 0(0) | 3(0) |
| Cuculiformes | Cuculidae | *Piaya cayana* | 5(4) | 4(1) | 0(0) | 0(0) | 9(5) |
| Piciformes | Picidae | *Picumnus fulvescens* | 2(0) | 2(0) | 0(0) | 3(0) | 7(0) |
| Passeriforme | Tyrannidae | *Pitangus sulphuratus* | 0(0) | 3(0) | 1(0) | 1(0) | 5(0) |
| Passeriforme | Polioptilidae | *Polioptila plumbea* | 4(1) | 5(3) | 2(0) | 7(2) | 18(6) |
| Passeriforme | Icteridae | *Procacicus solitarius* | 2(0) | 0(0) | 0(0) | 1(0) | 3(0) |
| Passeriforme | Thraupidae | *Schistochlamys ruficapillus* | 4(0) | 4(1) | 0(0) | 5(3) | 13(4) |
| Passeriforme | Thraupidae | *Sporophila albogularis* | 1(0) | 0(0) | 0(0) | 0(0) | 1(0) |
| Passeriforme | Furnariidae | *Synallaxis frontalis* | 0(0) | 0(0) | 0(0) | 1(0) | 1(0) |
| Passeriforme | Furnariidae | *Synallaxis scutata* | 0(0) | 1(0) | 0(0) | 0(0) | 1(0) |
| Passeriforme | Thraupidae | *Tachyphonus rufus* | 37(25) | 65(53) | 29(15) | 51(32) | 182(125) |
| Passeriforme | Thraupidae | *Tangara cayana* | 23(4) | 22(4) | 20(2) | 22(1) | 87(11) |
| Passeriforme | Thraupidae | *Tangara palmarum* | 0(0) | 2(1) | 0(0) | 1(0) | 3(1) |
| Passeriforme | Thraupidae | *Tangara sayaca* | 0(0) | 4(0) | 3(0) | 0(0) | 7(0) |
| Passeriforme | Thamnophilidae | *Taraba major* | 4(1) | 4(2) | 10(1) | 3(0) | 21(4) |
| Passeriforme | Thamnophilidae | *Thamnophilus doliatus capistratus* | 3(0) | 4(0) | 8(2) | 6(1) | 21(3) |
| Passeriforme | Thamnophilidae | *Thamnophilus pelzelni* | 0(0) | 1(0) | 0(0) | 0(0) | 1(0) |
| Passeriforme | Rhynchocyclidae | *Tolmomyias flaviventris* | 0(0) | 2(0) | 3(1) | 7(1) | 12(2) |
| Passeriforme | Troglodytidae | *Troglodytes musculus* | 1(0) | 0(0) | 2(0) | 0(0) | 3(0) |
| Trogoniformes | Trogonidae | *Trogon curucui* | 4(0) | 11(1) | 1(0) | 2(1) | 18(2) |
| Passeriforme | Turdidae | *Turdus amaurochalinus* | 104(17) | 35(2) | 17(2) | 8(2) | 164(23) |
| Passeriforme | Turdidae | *Turdus flavipes* | 8(2) | 0(0) | 4(1) | 1(0) | 13(3) |
| Passeriforme | Turdidae | *Turdus leucomelas* | 71(9) | 36(13) | 11(0) | 13(0) | 131(22) |
| Passeriforme | Turdidae | *Turdus rufiventris* | 1(1) | 0(0) | 0(0) | 0(0) | 1(1) |
| Passeriforme | Vireonidae | *Vireo chivi* | 5(2) | 0(0) | 4(3) | 1(0) | 10(5) |

Supplementary Table S2. Parasites lineages detected in the two years of sampling, with their respective genera/subgenera, codes in network drawings, and corresponding sequences in GenBank (P = *Plasmodium* and H = *Haemoproteus*). Lineages detected for the first time are in bold.

| **Code** | **Parasite Lineage** | **Genus** | **Rainy** | **Dry** | **Seq Genbank** |
| --- | --- | --- | --- | --- | --- |
| BAF03 | BAFLA03 | *P. plasmodium* | x |  | MK264393 |
| BAF04 | BAFLA04* | *P. plasmodium* | x | x | MK264394 |
| CAL01 | **CALON01** | *P. plasmodium* | x | x | MK291501 |
| COT01 | COTAL01 | *H. haemoproteus* | x | x | MK264395 |
| CPC57 | CPCT57 | *P. plasmodium* | x |  | MK264392 |
| DEN03 | DENPET03* | *P. plasmodium* | x | x | MK264396 |
| ELA01 | ELALB01 | *H. parahaemoproteus* | x | x | MK264397 |
| FOG01 | **FOGRI01** | *P. plasmodium* |  | x | MK291502 |
| FOM04 | **FOMEL04** | *P. plasmodium* | x |  | MK291503 |
| H012 | H012 | *P. plasmodium* | x |  | MK264398 |
| HES01 | **HESEL01** | *H. parahaemoproteus* |  | x | MK291504 |
| HYA01 | **HYAMA-01** | *P. plasmodium* |  | x | MK291505 |
| LEC02 | LECOR02* | *P. plasmodium* | x | x | MK264399 |
| NYM01 | **NYMAC01** | *H. parahaemoproteus* |  | x | MK291506 |
| PAD09 | PADOM09* | *P. plasmodium* | x | x | MK264400 |
| PAD11 | PADOM11 | *P. plasmodium* |  | x | MK264401 |
| PAD17 | PADOM17 | *P. plasmodium* | x |  | MK264402 |
| PAM01 | PAMIT01* | *P. plasmodium* | x | x | MK264403 |
| PAP03 | PAPOL03 | *H. parahaemoproteus* | x | x | MK264404 |
| POP01 | **POPLU01** | *P. plasmodium* | x |  | MK291507 |
| SocH3 | SocH3^†^ | *H. haemoproteus* | x | x | MK264405 |
| SocH4 | SocH4 | *H. haemoproteus* |  | x | MK264406 |
| TAR02 | **TARUF02**^†^ | *H. parahaemoproteus* | x | x | MH260577 |
| TUA01 | TUAMA01 | *P. plasmodium* | x | x | MK264407 |
| TUR02 | TURNUD02 | *P. plasmodium* | x |  | MK264408 |
| U12 | U12 | *P. plasmodium* | x |  | MK264409 |
| UN203 | UN203 | *H. parahaemoproteus* | x | x | MK264410 |
| VIR02 | VIREO02 | *H. parahaemoproteus* | x |  | MK264411 |

*Central parasite lineages in *Plasmodium* network; †Central parasite lineage in *Haemoproteus* network

Supplementary Table S3. List of infected birds species from which parasites lineages were identified, their codes in network drawings, families, and orders, sampled during two sampling years in a coastal ecosystem at Barreira do Inferno Rocket Launch Center - CLBI, Northeastern Brazil.

| **Code** | **Order** | **Family** | **Bird species** | **Year 1** | | **Year 2** | | **Total** |
| --- | --- | --- | --- | --- | --- | --- | --- | --- |
|  |  |  |  | **Rainy** | **Dry** | **Rainy** | **Dry** |  |
| Canlon | Passeriforme | Troglodytidae | *Cantorchilus longirostris* | 1 | 1 | 1 |  | 3 |
| Coefla | Passeriforme | Thraupidae | *Coereba flaveola** | 1 | 7 | 2 | 1 | 11 |
| Colpas | Columbiformes | Columbidae | *Columbina passerina*^†^ | 2 | 5 |  |  | 7 |
| Coltal | Columbiformes | Columbidae | *Columbina talpacoti* |  | 3 | 1 |  | 4 |
| Corpil | Passeriforme | Thraupidae | *Coryphospingus pileatus* |  | 1 |  |  | 1 |
| Cyacya | Passeriforme | Corvidae | *Cyanocorax cyanopogon* | 2 |  |  |  | 2 |
| Cycguj | Passeriforme | Vireonidae | *Cyclarhis gujanensis*^†^ | 3 | 4 | 1 |  | 8 |
| Elachi | Passeriforme | Tyrannidae | *Elaenia chilensis** | 4 |  | 3 |  | 7 |
| Elaspe | Passeriforme | Tyrannidae | *Elaenia spectabilis* |  |  | 1 |  | 1 |
| Forgri | Passeriforme | Thamnophilidae | *Formicivora grisea* | 1 | 1 | 1 |  | 3 |
| Herpec | Passeriforme | Thamnophilidae | *Herpsilochmus pectoralis* |  | 2 |  |  | 2 |
| Hersel | Passeriforme | Thamnophilidae | *Herpsilochmus sellowi* |  | 1 | 1 |  | 2 |
| Hylama | Passeriforme | Vireonidae | *Hylophilus amaurocephalus* |  |  |  | 1 | 1 |
| Lepver | Columbiformes | Columbidae | *Leptotila verreauxi* |  |  | 1 |  | 1 |
| Myityr | Passeriforme | Tyrannidae | *Myiarchus tyrannulus* |  | 1 |  |  | 1 |
| Neopal | Passeriforme | Pipridae | *Neopelma pallescens* |  |  | 1 |  | 1 |
| Nysmac | Galbuliformes | Bucconidae | *Nystalus maculatus* |  | 1 |  | 2 | 3 |
| Pacpol | Passeriforme | Tityridae | *Pachyramphus polychopterus* | 1 | 1 |  |  | 2 |
| Piacay | Cuculiformes | Cuculidae | *Piaya cayana* | 1 |  |  |  | 1 |
| Polplu | Passeriforme | Polioptilidae | *Polioptila plumbea* | 1 | 2 |  |  | 3 |
| Tacruf | Passeriforme | Thraupidae | *Tachyphonus rufus**^†^ | 20 | 40 | 6 | 11 | 77 |
| Tancay | Passeriforme | Thraupidae | *Tangara cayana* |  | 1 |  |  | 1 |
| Turama | Passeriforme | Turdidae | *Turdus amaurochalinus** | 3 |  |  |  | 3 |
| Turfla | Passeriforme | Turdidae | *Turdus flavipes* | 2 |  | 1 |  | 3 |
| Turleu | Passeriforme | Turdidae | *Turdus leucomelas* | 1 | 1 |  |  | 2 |
| Viroli | Passeriforme | Vireonidae | *Vireo chivi* |  |  | 1 |  | 1 |

*Host central species in *Plasmodium* network; †Host central species in *Haemoproteus* network.

Supplementary Table S4**.** Pairwise temporal comparisons of *β-*diversity of bird-parasites interactions. “*β_WN_*” represents the total *β-*diversity of interactions, “*β_OS_*” corresponds to temporal dissimilarity due to the rearrangement of interactions between species that co-occur at different times (i.e., rewiring), and “*β_ST_*” represents the dissimilarity of interactions caused by species turnover over time.

| **Pairwise comparisons** | | ***β_WN_*** | ***β_OS_*** | ***β_ST_*** |  |
| --- | --- | --- | --- | --- | --- |
| 1 | | Early rainy 1-Late rainy 1 | 1 | 1 | 0 |
| 2 | | Early rainy 1-Early rainy 2 | 1 | 1 | 0 |
| 3 | | Early rainy 1-Late rainy 2 | 0.89 | 0 | 0.89 |
| 4 | | Early rainy 1-Early dry 1 | 0.85 | 0.33 | 0.51 |
| 5 | | Early rainy 1-Late dry 1 | 0.93 | 0.67 | 0.26 |
| 6 | | Early rainy 1-Early dry 2 | 1 | 1 | 0 |
| 7 | | Early rainy 1-Late dry 2 | 1 | 1 | 0 |
| 8 | | Late rainy 1-Early rainy 2 | 0.91 | 0.67 | 0.25 |
| 9 | | Late rainy 1-Late rainy 2 | 1 | 1 | 0 |
| 10 | | Late rainy 1-Early dry 1 | 0.73 | 0.14 | 0.58 |
| 11 | | Late rainy 1-Late dry 1 | 0.83 | 0.5 | 0.33 |
| 12 | | Late rainy 1-Early dry 2 | 0.67 | 0 | 0.67 |
| 13 | | Late rainy 1-Late dry 2 | 0.85 | 0 | 0.85 |
| 14 | | Early rainy 2-Late rainy 2 | 1 | 1 | 0 |
| 15 | | Early rainy 2-Early dry 1 | 0.92 | 0.67 | 0.25 |
| 16 | | Early rainy 2-Late dry 1 | 0.78 | 0.57 | 0.21 |
| 17 | | Early rainy 2-Early dry 2 | 0.87 | 0.33 | 0.53 |
| 18 | | Early rainy 2-Late dry 2 | 0.88 | 0 | 0.88 |
| 19 | | Late rainy 2-Early dry 1 | 1 | 1 | 0 |
| 20 | | Late rainy 2-Late dry 1 | 0.89 | 0.5 | 0.39 |
| 21 | | Late rainy 2-Early dry 2 | 1 | 1 | 0 |
| 22 | | Late rainy 2-Late dry 2 | 1 | 1 | 0 |
| 23 | | Early dry 1-Late dry 1 | 0.85 | 0.5 | 0.35 |
| 24 | | Early dry 1-Early dry 2 | 0.71 | 0 | 0.71 |
| 25 | | Early dry 1-Late dry 2 | 0.73 | 0 | 0.73 |
| 26 | | Late dry 1-Early dry 2 | 0.88 | 0.33 | 0.54 |
| 27 | | Late dry 1-Late dry 2 | 0.88 | 0 | 0.88 |
| 28 | | Early dry 2-Late dry 2 | 0.6 | 0 | 0.6 |
| **Mean** | |  | **0.88** | **0.50** | **0.37** |
| **Percentage** | |  | **100%** | **57%** | **43%** |

Supplementary Table S5. Metrics of avian-parasite networks studied over two years in a tropical coastal ecosystem at Barreira do Inferno Rocket Launch Center - CLBI, Northeastern Brazil. Asterisks represent metric’s significance in comparison to the Patefield null model (n=999 randomizations).

| **Seasons** | **Modularity (Q)** | **Specialization (H^2^’)** | **Niche Overlap (Horn)** |
| --- | --- | --- | --- |
| Early Rainy 1 | 0.65* | 0.81* | 0.14 |
| Late Rainy 1 | 0.63 | 0.64* | 0.06 |
| Early Rainy 2 | 0.67 | 0.00 | 0.07 |
| Late Rainy 2 | 0.50 | 1.00* | 0.00 |
| Early Dry 1 | 0.65* | 0.86* | 0.04* |
| Late Dry 1 | 0.69* | 0.69* | 0.07* |
| Early Dry 2 | 0.19 | 0.85 | 0.5 |
| Late Dry 2 | 0.25* | 0.63* | 0.5 |
| **Mean** | **0.53** | **0.70** | **0.17** |

Supplementary Figure S1. Bayesian phylogenetic inference showing haemosporidian lineages detected in a bird community studied in a coastal ecosystem at Barreira do Inferno Rocket Launch Center - CLBI, Northeastern Brazil. Numbers located near branches indicate the Bayesian probability values. The length of the branches is illustrated according to the number of nucleotide changes (see scale). Acronyms that precede the names of lineages: p. = *Plasmodium* spp.; h.h. = *Haemoproteus* (*Haemoproteus*) spp.; h.p. = *H*. (*Parahaemoproteus*) spp. *Leucocytozoon* *caulleryi* represents the outgroup.

**

**

Supplementary Figure S2. Climatogram indicating monthly mean temperature and accumulated rainfall during the 24 sampling months in a coastal tropical ecosystem located at Barreira do Inferno Rocket Launch Center - CLBI, Northeastern Brazil.

**
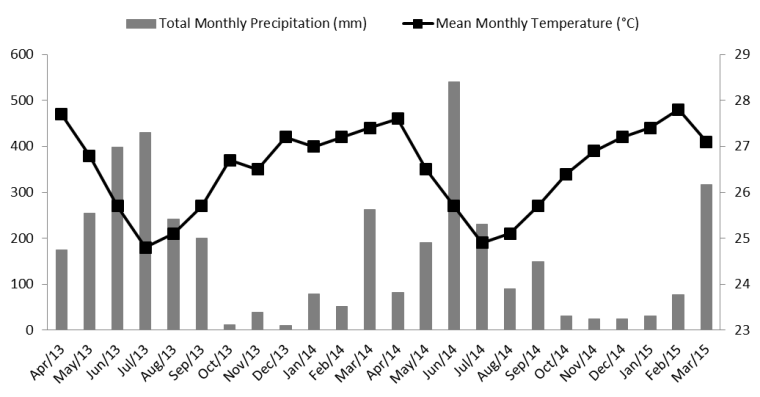
**
